# Deletion of Neuronal CuZnSOD Accelerates Age-Associated Muscle Mitochondria and Calcium Handling Dysfunction That Is Independent of Denervation and Precedes Sarcopenia

**DOI:** 10.3390/ijms221910735

**Published:** 2021-10-04

**Authors:** Yu Su, Dennis R. Claflin, Meixiang Huang, Carol S. Davis, Peter C. D. Macpherson, Arlan Richardson, Holly Van Remmen, Susan V. Brooks

**Affiliations:** 1 Department of Neurosurgery, First Affiliated Hospital, Sun Yat-sen University, Guangzhou 510080, China; syschol@umich.edu; 2 Department of Molecular and Integrative Physiology, University of Michigan, Ann Arbor, MI 48109, USA; csdav@umich.edu (C.S.D.); petercdm@med.umich.edu (P.C.D.M.); 3 Department of Surgery, Section of Plastic Surgery, University of Michigan, Ann Arbor, MI 48109, USA; claflin@umich.edu; 4 Department of Biomedical Engineering, University of Michigan, Ann Arbor, MI 48109, USA; 5 Department of Neurology, Second Xiangya Hospital, Central South University, Changsha 410011, China; mei.huang2020@outlook.com; 6 Department of Biochemistry and Molecular Biology, University of Oklahoma Health Sciences Center, Oklahoma City, OK 73104, USA; Arlan-Richardson@ouhsc.edu; 7 VA Medical Center, Oklahoma City, OK 73104, USA; holly-vanremmen@omrf.org; 8 Aging and Metabolism Research Program, Oklahoma Medical Research Foundation, Oklahoma City, OK 73104, USA; 9 Department of Physiology, Health Science Center, Oklahoma University, Oklahoma City, OK 73104, USA

**Keywords:** sarcopenia, oxidative stress, denervation, NADH, calcium

## Abstract

Skeletal muscle suffers atrophy and weakness with aging. Denervation, oxidative stress, and mitochondrial dysfunction are all proposed as contributors to age-associated muscle loss, but connections between these factors have not been established. We examined contractility, mitochondrial function, and intracellular calcium transients (ICTs) in muscles of mice throughout the life span to define their sequential relationships. We performed these same measures and analyzed neuromuscular junction (NMJ) morphology in mice with postnatal deletion of neuronal *Sod1* (i-mn-*Sod1*^-/-^ mice), previously shown to display accelerated age-associated muscle loss and exacerbation of denervation in old age, to test relationships between neuronal redox homeostasis, NMJ degeneration and mitochondrial function. In control mice, the amount and rate of the decrease in mitochondrial NADH during contraction was greater in middle than young age although force was not reduced, suggesting decreased efficiency of NADH utilization prior to the onset of weakness. Declines in both the peak of the ICT and force were observed in old age. Muscles of i-mn-*Sod1*^-/-^ mice showed degeneration of mitochondrial and calcium handling functions in middle-age and a decline in force generation to a level not different from the old control mice, with maintenance of NMJ morphology. Together, the findings support the conclusion that muscle mitochondrial function decreases during aging and in response to altered neuronal redox status prior to NMJ deterioration or loss of mass and force suggesting mitochondrial defects contribute to sarcopenia independent of denervation.

## 1. Introduction

Aging is associated with loss of skeletal muscle mass and impairments in function that are major contributors to frailty in the elderly [1,2]. These deficits, collectively referred to as sarcopenia, are observed in all animals that have been studied [2]. Although the fundamental causes of sarcopenia remain elusive, neuromuscular junctions (NMJ) displaying morphological abnormalities, including denervation, accumulate in muscles with aging [3,4], and elevations with aging in the production of reactive oxygen species by muscle mitochondria (mtROS) are also reported [5]. Thus, loss of muscle fiber innervation and impaired mitochondrial function are often implicated as contributors to sarcopenia progression [4,6,7], but mechanistic links between NMJ disruption, mitochondrial dysfunction and sarcopenia have not been established. 

Similar to normally aging mice, we previously reported degenerative changes at NMJs, elevated muscle mtROS generation, and loss of muscle mass and strength that develop by 8 months of age in mice lacking the major antioxidant enzyme copper zinc superoxide dismutase (CuZnSOD, *Sod1*^-/-^ mice) [8,9]. The denervation, mitochondrial dysfunction, and muscle atrophy in *Sod1*^-/-^ mice are all rescued by restoration of *Sod1* expression only in neurons (*SynTgSod1^-/-^* mice). The observation that denervation and mitochondrial dysfunction were concurrently corrected in *SynTgSod1^-/-^* mice [8,10] along with the known effects of denervation injury to cause dramatic increases in muscle mtROS [11,12,13] suggest that mitochondrial dysfunction in *Sod1^-/-^* mice is secondary to NMJ degeneration [8]. Finally, postnatal deletion of *Sod1* from only neurons results in accelerated age-associated loss of muscle mass and force and more extensive denervation at advanced age compared with wild type (WT) mice [14]. Collectively, these studies suggest a model in which loss of redox homeostasis in motor neurons triggers NMJ degeneration and denervation leading to impaired mitochondrial function and muscle atrophy and weakness, but this sequence of events has not been directly examined. 

In addition to impairments in mitochondrial respiration in aged muscle, calcium handling by the sarcoplasmic reticulum (SR) is also impaired [15], while mice with overexpression of the antioxidant catalase in mitochondria show preservation of SR calcium handling into old age [16] supporting physiological links between mitochondria and SR. Mitochondria and SR are also linked physically via the so-called mitochondrial associated membranes (MAM) [17], and this close spatial relationship allows mitochondria to rapidly buffer calcium released from the SR. Finally, calcium impacts mitochondrial function via regulation of the activity of enzymes of mitochondrial respiration [18]. 

To provide insights into the complex relationships between mitochondria, calcium, and sarcopenia, we studied muscle contractile properties throughout life, along with calcium handling, and mitochondrial function. In addition, using our mouse model with postnatal *Sod1* deletion from motor neurons (i-mn-*Sod1*^-/-^ mice) and control littermates, we tested whether alterations in redox homeostasis in motor neurons triggers muscle mitochondrial and SR dysfunction and determined the relationship of these deficits to loss of NMJ innervation.

## 2. Results

### 2.1. Lumbrical Muscle Contractile Properties Deteriorate with Aging, but Changes Occur Asynchronously for Different Parameters

**Figure 1:**
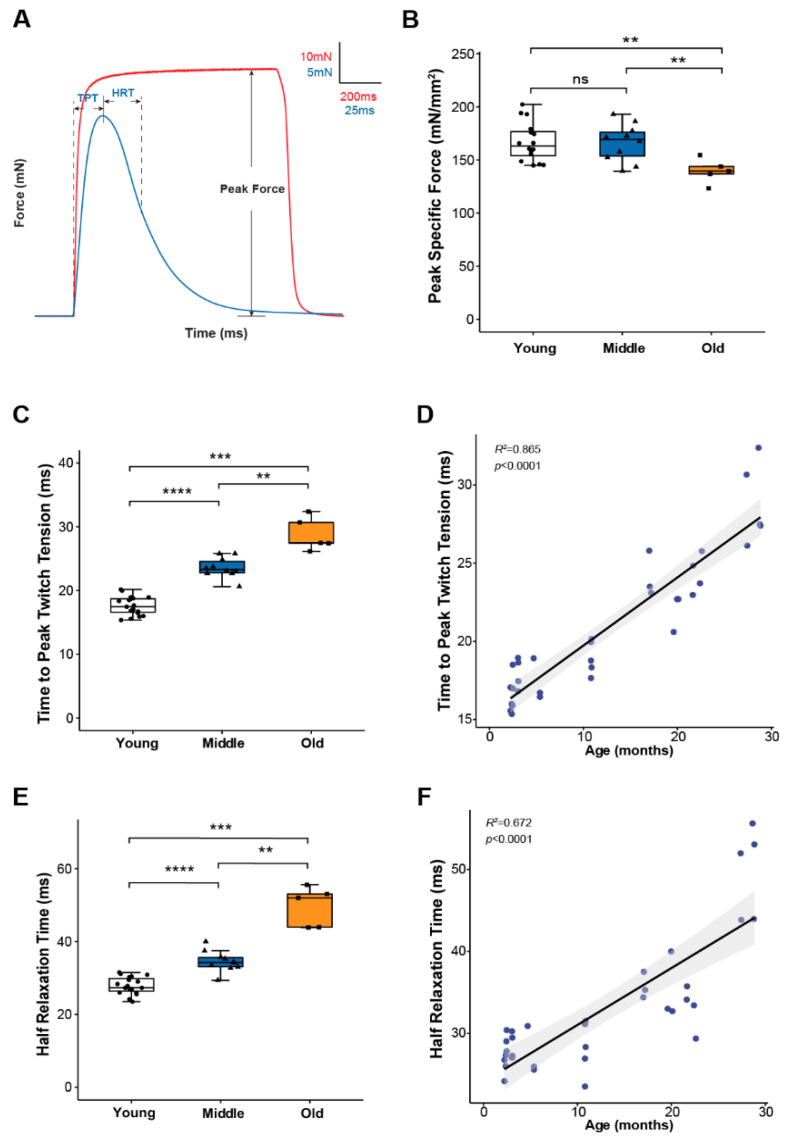
Lumbrical muscle contractile properties. (**A**) Representative traces of force generation during twitch (blue) and tetanic (red) contractions, with parameters used to describe muscle contractile properties annotated on the graph. Data shown for (**B**) peak specific force, (**C**) time to peak twitch tension (TPT), and (**E**) half relaxation time (HRT) for lumbrical muscles from young (n = 18, white, cricle), middle (n = 10, blue, triangle) and old (n = 5, orange, square) aged wild type mice. Specific force was calculated by normalizing the maximum isometric tetanic force by maximum muscle cross-sectional area. Additionally, shown are correlations between (**D**) age and TPT and (**F**) age and HRT analyzed by Pearson correlation (TPT: R^2^ = 0.865, *p* < 0.0001; HRT: R^2^ = 0.672, *p* < 0.0001). Data in (**B**,**C**,**E**) are presented as individual data points with box plots and error bars indicating the minimum, first quartile, median, third quartile, and maximum number of the dataset. Data were analyzed by one-way ANOVA, ** *p* < 0.01, *** *p* < 0.001, **** *p* < 0.0001.

To study skeletal muscle physiological parameters during aging, we used mouse hind paw lumbrical muscles due to the significant advantage provided by their small size allowing the application of fluorescence microscopy to monitor calcium handling and mitochondrial function in real time during contractions [8,19]. We first examined lumbrical muscle contractile properties throughout life and report data for maximum force generation and twitch contraction times for young (2–11 months), middle-aged (15–23 months) and old (25–29 months) mice (Figure 1). The results show a decoupling between the age-associated changes in force generating capacity and contraction kinetics. Lumbrical muscles displayed no significant age-associated loss of mass and no decrease in peak specific force until old age (Figure 1B) while contractile twitch times increased progressively throughout the life span. Both time to peak twitch tension (TPT) (Figure 1C) and half relaxation time (HRT) (Figure 1E) increased by 30-40% between young and middle-aged mice with similar further increases observed between middle and old age. In fact, age explained 86% and 67% of the variation in TPT and HRT, respectively (Figure 1D,F). Since twitch times are determined by SR calcium release, calcium buffering, and reuptake by the SR, these findings suggest that calcium handling and/or calcium sensitivity are significantly affected by age with changes expected to be evident by middle age prior to the presentation of sarcopenic muscle atrophy and weakness.

### 2.2. Skeletal Muscle Calcium Handling Function Starts to Decline in Middle Age

**Figure 2:**
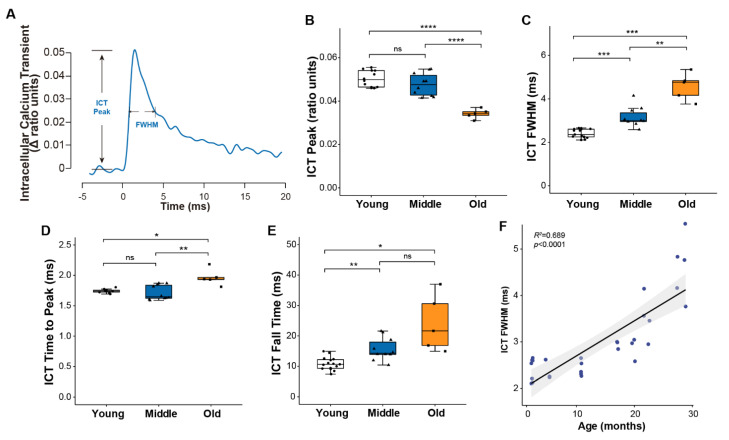
Muscle calcium handling properties. (**A**) Representative traces of intracellular calcium transient reported by mag-fura-2 during a single twitch contraction, with parameters used to describe calcium handling properties annotated on the graph. Data are shown for (**B**) the peak of the intracellular calcium transient (ICT peak), (**C**) time during which the ICT remains at or above its half maximum width (full width at half-maximum, ICT FWHM), (**D**) time for ICT transient increased to its peak, and (**E**) time for ICT transient to decrease from 90% to 10% of its maximum for lumbrical muscles of young (n = 10, white, circle), middle (n = 10, blue, triangle) and old (n = 5, orange, square) aged wild type mice. Additionally, shown is (**F**) the correlation between age and ICT FWHM analyzed by Pearson correlation (R^2^ = 0.689, *p* < 0.0001). Data in (**B**–**E**) are presented as individual data points with box plots and error bars indicating the minimum, first quartile, median, third quartile, and maximum number of the dataset. Data were analyzed by one-way ANOVA, * *p* < 0.05, ** *p* < 0.01, *** *p* < 0.001, **** *p* < 0.0001.

To examine age-associated changes in calcium handling, we monitored intracellular calcium transients (ICT) using the low affinity calcium-sensitive dye mag-fura-2 (Figure 2A). Consistent with the similar force generating capacity for muscles of young and middle-aged mice, peak calcium levels were not different between the two age groups, while a roughly 30% decline in the peak of the calcium transient by old age (Figure 2B) suggests the decrease in specific force generation by old muscles may be driven at least in part by reduced calcium release. The progressive slowing of the twitch throughout life was mirrored by a longer duration calcium transient quantified by the full width at half maximum (FWHM) (Figure 2C), ICT time to peak (Figure 2D) and ICT fall time (Figure 2E), all of which showed consistent and continued increases throughout life. Over the entire range studied, 69% of the variation in the ICT FWHM was explained by age (Figure 2F).

### 2.3. Skeletal Muscle Mitochondria Function Is Diminished by Middle Age

**Figure 3:**
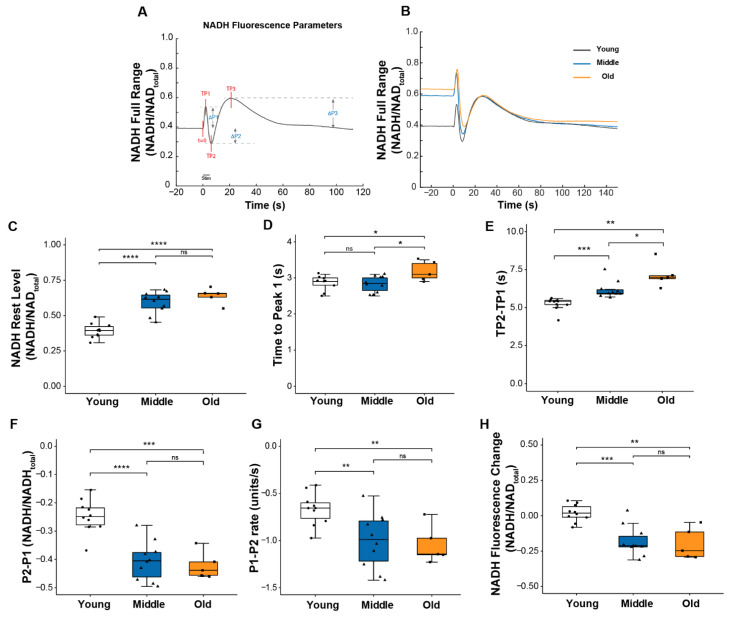
NADH fluorescence response to a 5s tetanic contraction. (**A**) A representative trace of NADH fluorescence dynamics illustrating parameters used to quantify the NADH fluorescence response scaled to maximum and minimum NADH levels. Times to each peak (TP1, TP2, TP3) were measured relative to *t* = 0; peak amplitudes (ΔP1, ΔP2, ΔP3) were measured relative to pre-contraction resting level. (**B**) Representative NADH fluorescence records for lumbrical muscles of young (black), middle (blue), and old (orange) aged mice. Data are shown for (**C**) basal NADH level prior to contraction, (**D**) time for NADH fluorescence increase to the 1st peak with contraction, (**E**) time between NADH fluorescence peaks P1 and P2 (TP2 minus TP1), (**F**) amplitude of changes in NADH fluorescence between P1 and P2, (**G**) rate of change in NADH fluorescence between P1 to P2, and (**H**) NADH level recorded 100 s after the end of the contraction relative to rest level for lumbrical muscles of young (n = 10, white), middle (n = 10, blue) and old (n = 5, orange) aged wild type mice. Data in (**C**–**H**) are presented as individual data points with box plots and error bars indicating the minimum, first quartile, median, third quartile, and maximum number of the dataset. Data were analyzed by one-way ANOVA, * *p* < 0.05, ** *p* < 0.01, *** *p* < 0.001, **** *p* < 0.0001.

Mitochondrial respiration activity is regulated in part by calcium, and we previously suggested that changes in intracellular calcium might be one of the contributors to the impaired mitochondrial function we observed in *Sod1*^-/-^ mice [8]. Thus, we were interested in whether the age-associated changes observed here in calcium handling were concurrent with changes in mitochondrial function. To address this question, we assessed mitochondrial function during contraction by monitoring the NADH fluorescence emitted in response to excitation of the muscle with ultraviolet light as previously described [8,19]. The fluorescence reflects NADH levels determined by the balance between NADH production and utilization and displays a highly reproducible pattern of oscillations as shown in Figure 3A,B. These oscillations reveal the dynamics of mitochondrial respiratory activity in the context of normal physiological function. Three distinct peaks can be defined relative to resting NADH level (Figure 3A). The first peak, P1, is caused primarily by NADH production from glycolysis [20]. The change in direction from an increase in fluorescence to a decrease between P1 and P2 is mainly caused by the net utilization of NADH through oxidative phosphorylation (OXPHOS) in response to contractile activity. Finally, the post-contraction increase in fluorescence from P2 to P3 results from delayed activation of the TCA cycle regenerating NADH [21].

Several notable age-associated changes were observed in the dynamics of the NADH response to contractions that support the development of impairments in mitochondrial function by middle age. As shown in Figure 3E–G, both the magnitude and the rate of change in NADH between P1 and P2 were significantly greater for muscle of middle-aged compared with young mice, although force generation was unchanged. Together, these findings indicate a reduction in the efficiency of NADH utilization. Further declines in these values were not seen between middle and old age, but the time to reach P2 (TP2-TP1) did continue to increase into old age (Figure 3E), suggesting a delay in reaching a sufficient level of post-contraction regeneration of NADH to exceed its further utilization. Finally, in contrast to young muscles in which the NADH oscillatory response was resolved within approximately 1 min of the cessation of contraction, for both middle and old age groups, NADH levels remained low relative to resting levels for a prolonged period of time following the tetanic contraction (Figure 3H), perhaps owing to an elevated basal NADH level in those age groups (Figure 3B,C). Finally, for muscles of old mice compared to young or middle-aged mice, the time to reach P1 (TP1), the point at which NADH utilization through oxidative phosphorylation exceeds its production by glycolysis, was extended (Figure 3D) reflecting a decreased sensitivity of the OXPHOS machinery to energy demand. Collectively, these findings show mitochondrial dysfunction in lumbrical muscles by middle age, prior to any reduction in the peak of the ICT, although the ICT was slightly prolonged.

### 2.4. Knock Down of Sod1 in Motor Neurons Induces Post-Synaptic Changes in Myofiber Physiology without Evidence of Denervation

**Figure 4:**
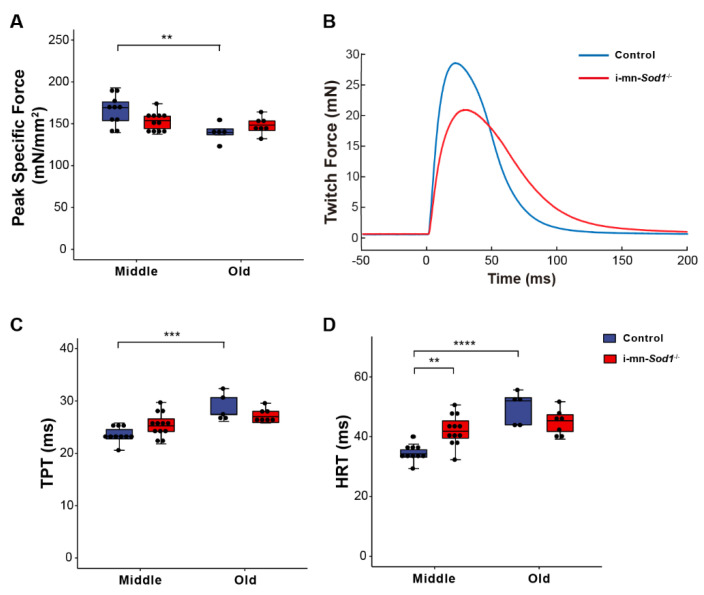
Comparison of lumbrical muscle contractile properties between control and i-mn-*Sod1*^-/-^ mice. Data are shown for (**A**) peak specific force for muscles of middle-aged and old control (blue) and i-mn-*Sod1*^-/-^ (red) mice. Additionally, shown are (**B**) representative twitch contractions for muscles of control (blue) and i-mn-*Sod1*^-/-^ (red) mice at middle-age as well as data for (**C**) time to peak twitch tension (TPT), (**D**) half relaxation time (HRT) for muscles of middle- and old-aged control (blue; n = 10 for middle age, n = 5 for old age) and i-mn-*Sod1*^-/-^ (red; n = 12 for middle age, n = 7 for old age) mice. Data in A, C, and D are presented as individual data points (circle) with box plots and error bars indicating the minimum, first quartile, median, third quartile, and maximum number of the dataset. Data were analyzed by two-way ANOVA, ** *p* < 0.01, *** *p* < 0.001, **** *p* < 0.0001.

We recently showed that postnatal deletion of *Sod1* from motor neurons (i-mn-*Sod1*^-/-^ mice) resulted in an acceleration of age-associated muscle atrophy such that nearly all muscles examined showed reduced mass by middle age, while in old age, denervation was more extensive in i-mn-*Sod1*^-/-^ mice than controls [14]. In the present study, we extended these studies to examine mitochondrial function and calcium handling in muscles of middle-aged and old i-mn-*Sod1*^-/-^ mice to determine whether the age-associated defects observed for these post-synaptic cellular functions were accelerated or intensified by the loss of neuronal *Sod1* and whether denervation was an essential component of the changes. While WT muscles showed a decline in peak isometric specific force between middle and old age (Figure 1B), muscles of i-mn-*Sod1*^-/-^ mice showed an earlier decline, with the force generating capacity reduced by middle age to a level that was not different from the old control mice (Figure 4A). Muscles of i-mn-*Sod1*^-/-^ mice also showed slowing of twitch times by middle age to values not different from old control mice (Figure 4C,D), while control muscles showed slowing of twitch contractions between middle and old age (Figure 4C,D) as observed for aging WT mice (Figure 1C,E). Collectively, these data show that deletion of *Sod1* from motor neurons accelerates the development of age-associated declines in muscle contractile properties. We next questioned whether the deficits in muscle function in i-mn-*Sod1*^-/-^ mice are associated with mitochondrial and calcium handling dysfunction as observed for WT mice.

**Figure 5:**
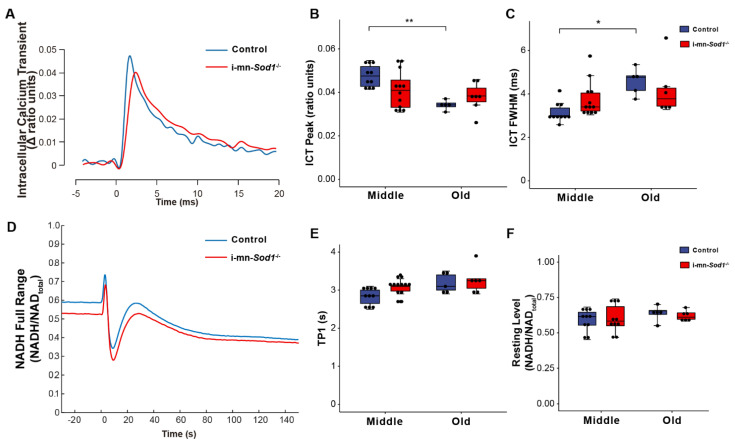
Comparison of calcium handling and mitochondrial function between control and i-mn-*Sod1*^-/-^ mice. Representative traces are shown for (**A**) intracellular calcium transients (ICT) and (**D**) NADH fluorescence response to a tetanic contraction for lumbrical muscles of control (blue) and i-mn-*Sod1*^-/-^ (red) mice at middle-age. Data are shown for (**B**) the peak of the intracellular calcium transient (ICT peak), (**C**) time during which the ICT remains at or above its half maximum width (full width at half-maximum, ICT FWHM), (**E**) time for NADH fluorescence increase to the 1st peak with contraction, and (**F**) basal NADH level prior to contraction for control (blue; n=10 for middle age, n=5 for old age, blue) and i-mn-*Sod1*^-/-^ (red; n = 12 for middle age, n = 7 for old age) mice. Data in (**B**,**C**,**E**,**F**) are presented as individual data points with box plots and error bars indicating the minimum, first quartile, median, third quartile, and maximum number of the dataset. Data were analyzed by two-way ANOVA, * *p* < 0.05, ** *p* < 0.01.

If mitochondrial dysfunction contributes to sarcopenia, muscles of i-mn-*Sod1*^-/-^ mice should display mitochondrial dysfunction by middle age. Although muscles of i-mn-*Sod1*^-/-^ mice showed no significant differences from controls by two-way ANOVA for changes in NADH fluorescence in response to contraction (Figure 5D–F), the lack of difference largely reflects the diminished mitochondrial function already present by middle age in control mice. In addition, a trend for prolongation of TP1 was observed for muscles of middle-aged i-mn-*Sod1*^-/-^ mice compared with age-matched controls (Figure 5E). The 10% larger value for TP1 for mitochondria in i-mn-*Sod1*^-/-^ compared with control muscles is statistically significant by *t* test and indicative of an accelerated degeneration of mitochondria in i-mn-*Sod1*^-/-^ mice. Muscles of i-mn-*Sod1*^-/-^ mice also showed some evidence for an acceleration of the aging effects on calcium handling. Large variation in the properties of the ICT displayed for muscles of middle-aged i-mn-*Sod1*^-/-^ mice prevented significance when analyzed by two factor ANOVA; however, when i-mn-*Sod1*^-/-^ and control muscles were compared by *t*-test, the differences in both ICT peak and ICT FWHM were highly significant for middle-aged mice. The middle-aged i-mn-*Sod1*^-/-^ mice appear to be at a threshold for worsening defects in calcium handling. 

**Figure 6:**
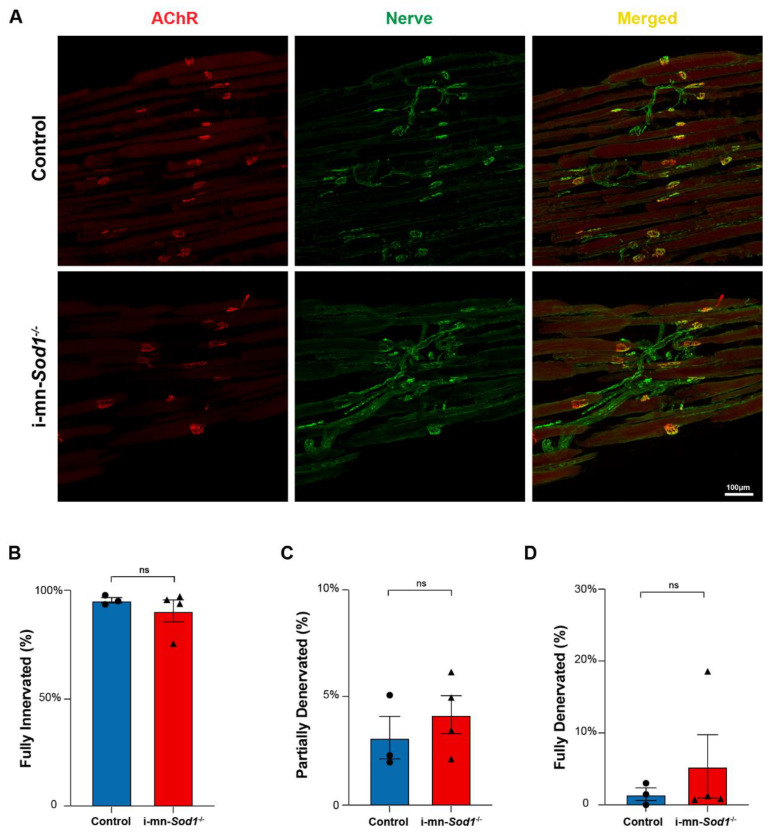
NMJ morphology in control and i-mn-*Sod1*^-/-^ mice. (**A**) Representative immunofluorescent images of NMJs in lumbrical muscles of 20-month-old control and i-mn-*Sod1*^-/-^ mice. Muscles were stained for nerve with anti-βIII Tubulin antibody (green) and acetylcholine receptors, AChR with BTX (red) to visualize pre- and post-synaptic structure, respectively. Scale bar (100 µm) is labeled in image. Data are shown for the number of (**B**) fully innervated, (**C**) partially denervated, and (**D**) fully denervated endplates for control (n = 3, blue, circle) and i-mn-*Sod1*^-/-^ (n = 4, red, triangle) mice. Data in (**B**–**D**) are presented as mean+SEM including individual data points and analyzed by Student’s *t*-test. There were no differences between any groups.

Impairments in skeletal muscle mitochondrial function have previously been hypothesized to be a consequence of denervation and/or degenerative changes at the NMJ [8]. Muscles of i-mn-*Sod1*^-/-^ mice display significantly greater levels of NMJ degeneration and denervation than controls in gastrocnemius muscles at 24 months of age. Here, we determined whether the innervation status was a contributor to our findings of impaired mitochondrial and calcium handling function in lumbrical muscles of middle-aged i-mn-*Sod1*^-/-^ mice (Figure 5). NMJ structure was examined in lumbrical muscles from middle-aged i-mn-*Sod1*^-/-^ and control mice using immunofluorescent staining techniques (Figure 6A). Analysis of these images showed that muscles in both i-mn-*Sod1*^-/-^ mice and controls had largely intact NMJ morphology with unbroken pretzel-like arrangements of acetylcholine receptors and no significant loss of overlap between pre- and post-synaptic structures (Figure 6B–D). These findings indicate that the age-associated defects in mitochondrial function and calcium handling observed in middle-aged i-mn-*Sod1*^-/-^ and control mice develop at an age when there was no evidence of NMJ deterioration or denervation.

**Figure 7:**
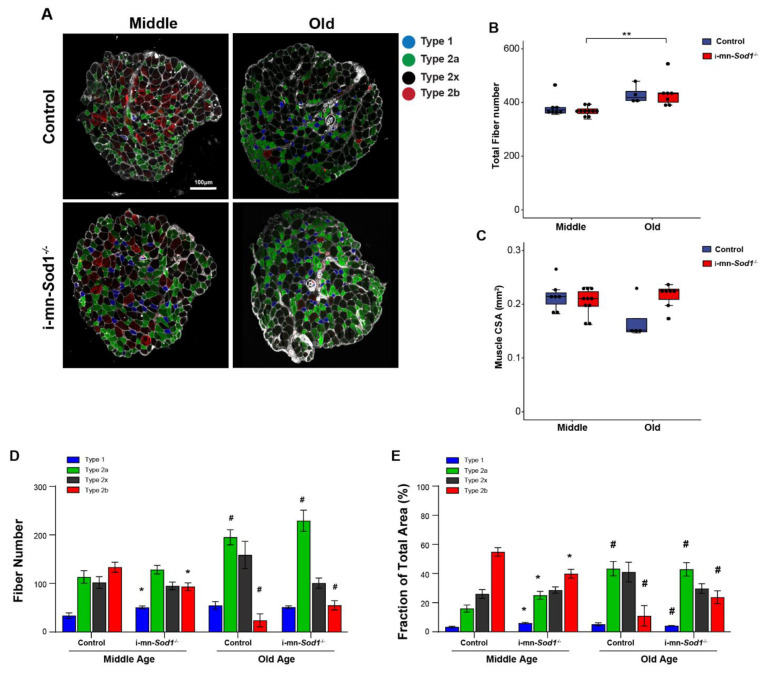
Fiber type composition in lumbrical muscle between control and i-mn-*Sod1*^-/-^ mice. (**A**) Representative immunofluorescence images of lumbrical muscle cross sections for control and i-mn-*Sod1*^-/-^ mice at middle- and old-age. Fiber types are represented in different pseudo-color as type 1 (blue), type 2a (green), type 2x (black), type 2b (red). Scale bar (100 µm) is labeled in image. Data are shown for (**B**) total fiber number and (**C**) total lumbrical muscle cross-sectional area for muscles of controls (blue; n = 7 for middle age, n = 4 for old age) and i-mn-*Sod1*^-/-^ mice (red; n = 10 for middle age, n = 7 for old age) and are presented as individual data points with box plots and error bars indicating the minimum, first quartile, median, third quartile, and maximum number of the dataset. These data were analyzed by two-way ANOVA, ** *p* < 0.01. Additionally, shown are (**D**) the number of each type of fiber and (**E**) the fraction of the total muscle cross-sectional area occupied by each type of fiber in lumbrical muscles from control and i-mn-*Sod1*^-/-^ mice at middle and old-age. Data are presented as means+SEM and analyzed by two-way ANOVA, Turkey’s multiple comparisons test. * *p* < 0.05 compared to control littermates and # *p* < 0.05 compared to same genotype mice at middle-age. Sample sizes for control mice are n = 7 for middle age and n = 4 for old age and n = 10 for middle age and n = 7 for old age for i-mn-*Sod1*^-/-^ mice.

Finally, we asked whether disruption of redox homeostasis in motor neurons would induce shifts in muscle fiber types. As shown by immunofluorescent staining for myosin heavy chain isoforms in cross sections of lumbrical muscles (Figure 7A), i-mn-*Sod1*^-/-^ mice showed a shift of muscle fiber type from glycolytic to oxidative, as the number of type 2b fibers was 30% lower and the number of type 1 fibers was more than 50% greater in muscles of middle-aged i-mn-*Sod1*^-/-^ mice compared to age-matched controls (Figure 7D). The shift in fiber type distribution resulted in significant differences between i-mn-*Sod1*^-/-^ and control mice in the fraction of the cross section occupied by oxidative and glycolytic fibers (Figure 7E). The percent of the cross section comprised of type 2b fibers was nearly 30% lower in muscles of middle-aged i-mn-*Sod1*^-/-^ mice compared with age-matched controls, while the percent comprised of type 1 and type 2a fibers was 80% and 56% higher, respectively (Figure 7E). This pattern of fiber type shifting was similar to that observed during normal aging, as seen by comparing muscles of old and middle-aged control mice which showed an increase with age in the number of oxidative fibers and a decrease in glycolytic fibers (Figure 7D,E). Interestingly, with increasing age the fiber type composition of both genotypes was quite comparable (Figure 7D,E).

## 3. Discussion

Degeneration of NMJs is a prominent feature of skeletal muscle during aging [4], and deterioration in the communication between motor neurons and muscle fibers has long been implicated in the onset and progression of sarcopenia [22,23]. Mitochondrial dysfunction is also an accepted hallmark of aging in numerous tissues and often hypothesized as a causative factor in age-associated decline in function [24,25]. Skeletal muscle attracts much attention in this regard due to the high concentration of mitochondria and high energy demand [6]. Previous work from our group supports both the importance of neuronal and NMJ function for the maintenance of muscle during aging [4,8] as well as strong associations between innervation and mitochondrial function [13]. Specifically, we showed in a model of accelerated neuromuscular aging resulting from deficiency in the major antioxidant enzyme CuZnSOD (*Sod1*^-/-^ mice) that skeletal muscle loss in early adulthood was associated with NMJ degeneration and mitochondrial impairments, while restoring NMJ integrity or protecting from mitochondrial dysfunction in this model preserved muscle structure and function until late life [8,10,26,27]. Since preservation of innervation, mitochondrial function and muscle size and strength were all coincident in these models, whether the detrimental phenotypes in the muscle are simply secondary to NMJ degeneration and denervation remained unclear. Recently, we showed that postnatal deletion of *Sod1* in neurons alone (i-mn-*Sod1*^-/-^ mice) led to muscle atrophy earlier in life than in wild type mice and more extensive denervation in i-mn-*Sod1*^-/-^ mice in old age [14], but links between NMJ degeneration, mitochondrial function, and muscle loss were not established. In the present study, through examination of mitochondrial function, excitation contraction (EC)-coupling, sarcopenia, and denervation throughout life in WT and i-mn-*Sod1*^-/-^ mice, we provide insights into the sequential nature of the age-associated changes in these phenotypes.

The primary findings of the present study were that mitochondrial and EC-coupling dysfunction were apparent prior to evidence of atrophy, weakness, or degenerative changes at NMJs. While maximum specific force for muscles of middle-aged WT mice was maintained at values not different from young mice, mitochondrial function was already diminished as were kinetic aspects of calcium handling. Furthermore, i-mn-*Sod1*^-/-^ mice showed evidence for an acceleration of the age-associated declines in both muscle mitochondrial function and calcium handling resulting from the deletion of neuronal *Sod1*. In all cases, the impairments in post-synaptic cellular functions were observed at an age when no morphological evidence of NMJ degeneration or loss of innervation was observed. These findings extend our understanding of the progression of sarcopenic changes during aging and demonstrate that alterations in neuronal redox homeostasis can impact muscle mitochondrial function through mechanisms independent of denervation.

Muscle mitochondria degenerate morphologically as well as functionally during aging. Compared to young control mice, muscles of old mice display intermyofibrillar mitochondria that are longer and more branched while subsarcolemmal mitochondria are larger and also more elongated [28]. The respiratory capacity of mitochondria isolated from old muscle also show impairments [29]. One consideration with the common means of measuring mitochondrial function is the potential for damaging mitochondria during isolation procedures that may induce alterations in the function [30,31]. Differential effects of the isolation procedures on muscle fibers with differences in the structure of their mitochondrial networks are easy to imagine. In the current study, we examined mitochondrial function by monitoring NADH dynamics in mitochondria of intact contracting muscles in real time. This method leaves mitochondria undisturbed in their location in the cell, in other intracellular interactions, and in fluctuations that may occur in the composition of the cytosol during physiological function. Our finding that mitochondria showed significant deleterious changes in the dynamics of NADH before evidence of muscle atrophy or weakness is consistent with a mechanistic link between defects in mitochondrial function and the onset of sarcopenia [32,33,34]. Dysfunctional mitochondria could promote the development of muscle atrophy and weakness through multiple pathways, such as ROS production and oxidative damage [33,35], pro-apoptotic signaling [36], and/or impaired coupling and energy provision [37,38]. Our prior work found no increase in ROS generation by muscle mitochondria from i-mn-*Sod1*^-/-^ mice compared with control, although there was evidence of reduced expression of important mitochondrial genes in muscles of i-mn-*Sod1*^-/-^ mice as well as higher levels of oxidative damage at both 10 and 20 months of age, prior to a significant increase in denervated NMJs [14]. Increased levels of oxidative stress have been clearly shown to induce pathology and dysfunction in skeletal muscle [9,39,40], but even transient and subtle changes in reactive oxygen species (ROS) act as important mediators to regulate cell physiology [41,42].

The observation in the current study that muscle mitochondrial dysfunction appears to precede calcium handling dysfunction in normal aging is novel. Mitochondrial respiration is partly dependent on calcium levels as calcium is required in the mitochondrial matrix to stimulate oxidative metabolism through the regulation of three dehydrogenases in the TCA cycle [43]. Thus, it has been proposed that the contraction-induced calcium transient is one determinant of skeletal muscle mitochondrial function [18], and our prior investigations implicate a role for the alterations in the calcium transient in the mitochondrial dysfunction in muscles of *Sod1*^-/-^ mice [8]. In particular, the work of Qaisar et al. [44] showing that pharmacological restoration of SR calcium handling in muscles of *Sod1*^-/-^ mice reduced mitochondrial ROS production, although mitochondrial respiratory capacity was not assessed. The findings of the present study showing impairments in mitochondrial function at middle age while the peak of the calcium transient remains unchanged suggests that mitochondrial defects may by a driver for the development of age-associated impairments in calcium handling rather than vice versa. Skeletal muscle calcium handling is dominated by its release and uptake by the SR, but mitochondria also serve as a significant calcium sink in myocytes [18] as evidenced by experiments showing that inhibition of calcium uptake by mitochondria has the effect of increasing cytosolic calcium concentration [45]. Mitochondria can also regulate calcium transients via rapid uptake calcium at the mouth of calcium channels on the mitochondria associated membrane (MAM) [46]. Although there were no differences in the present study in the peak of the calcium transient between young and middle-aged mice, our observation that the width of the transient is broadened by middle age may implicate impaired ability of mitochondria to buffer calcium as an early change in mitochondrial function. The complexity of the crosstalk between mitochondria and SR, especially during aging or in response to denervation clearly warrants additional investigation.

One surprising result of our study is that while i-mn-*Sod1*^-/-^ mice exhibited some degenerative changes by middle age, the NMJ structure remains largely intact. These findings are consistent with our prior studies of gastrocnemius muscles in which i-mn-*Sod1*^-/-^ mice only showed substantial denervation and degenerative changes in NMJ structure after approximately 24 months of age [14]. Although we found no evidence of denervation in lumbrical muscles of middle-aged mice, our observation of fiber type shifting in middle-aged i-mn-*Sod1*^-/-^ mice is likely indicative of remodeling of innervation [47]Fiber types are described based on two distinct physiological parameters, speed of contraction and resistance to fatigue [48]. The changes we observed in the lumbrical muscles in the current study indicate that both normally aging mice and mice with alterations in neuronal redox homeostasis display the general pattern of shifts to a slower more oxidative fiber type composition consistent with prior reports [49]. Slow twitch muscles produce fewer mitochondrial ROS [50] and display lower capacity for mitochondrial calcium retention [6] than fast twitch muscles. Moreover, calcium transients vary significantly with fiber type, with fast fibers showing higher peak calcium levels and shorter durations than slow fibers. Thus, our data on changes in mitochondrial function and calcium handling with aging and/or altered neuronal redox status likely reflect a combination of intrinsic differences as well as being the consequence of fiber type shifting [51].

While recent reports have emphasized NMJ degeneration as a focal point in sarcopenia progression [52], skeletal muscle atrophy may also be driven in part by a reduction in motor neuron number with aging. This possibility is supported by the observations that lumbar spinal cords from a rat model of sarcopenia displayed a 27% reduction in the motor neuron pool between young and old ages [53], and in humans over 60 years old, spinal cord staining and quantitative electromyography showed age-associated declines in motor neuron number [54,55]. However, others report degenerative changes at NMJs with no evidence of motor neuron loss [56,57]. Regardless of the importance of central vs. peripheral changes, neurons like myocytes are particularly susceptible to redox dysregulation due to their large size and high consumption of oxygen. ROS can damage neurons [58], and the ability of neuronal cells to maintain their normal redox state diminishes during aging [59]. Substantial evidence supports the critical importance of redox homeostasis in normal functioning of the nervous system [60], and redox dysregulation contributes to numerous neurodegenerative diseases [61]. Moreover, declining neuronal mitochondrial respiratory capacity may also contribute to NMJ degeneration, as Hayes et al. have reported an age-dependent loss of mitochondria from motor neurons in *Sod1*^-/-^ mice [62]. Our data showing that specific deletion of neuronal *Sod1* induced an acceleration of the age-associated deficits in muscle mitochondria and calcium handling function prior to NMJ deterioration suggest relevant changes in redox-dependent signals from neurons to muscle that alter NMJ and muscle fiber function prior to structural evidence of disruption of innervation.

## 4. Materials and Methods

### 4.1. Animals

A combination of male and female mice was used in this study. All mice were on a C57BL/6 background. The wild type mice were bred housed to the appropriate ages in our colonies. The generation of i-mn-*Sod1*^-/-^ mice was described in detail previously [14]. Briefly, i-mn-*Sod1*^-/-^ mice were generated by breeding *Sod1*^flox/flox^ mice to *Sod1*^flox/flox^ SlickCre positive mice to generate control (*Sod1*^flox/flox^) and i-mn-*Sod1*^-/-^ mice (*Sod1*^flox/flox^ SlickCre positive) after tamoxifen administration. The SlickH Cre mice were purchased from Jackson Laboratories (Bar Harbor, ME USA) and *Sod1*^flox/flox^ mice were generated by our group [26]. To induce motor-neuron deletion of *Sod1*, intraperitoneal injections of tamoxifen were administrated (60 mg/kg body weight) to *Sod1*^flox/flox;SlickHCre^ (i-mn-*Sod1*^-/-^) with 2 rounds of 5 consecutive days at the age of 2–4 months old. Littermate *Sod1*^flox/flox;SlickHwt^ mice were similarly administered tamoxifen to serve as controls. All mice were housed 3–4/cage on a 12 h dark/light cycle and maintained under specific pathogen free conditions and provided with food and water ad libitum. Mice were sacrificed at various ages as described for experimentation. Prior to sacrifice, mice were anesthetized with an intraperitoneal injection of tribromoethanol (Avertin, 400 mg/kg) and mice hind paws were removed and immersed in Tyrode’s solution (composition below). After removal of the hind paws, mice were administered an overdose injection of the anesthetic followed by a bilateral thoracotomy to ensure euthanasia. We classified mice 2- to 11-months-old as young, 15- to 23-months-old as middle aged, and 25- to 29-months-old as old. Numbers of animals used in this study are as follows: n = 18 for young WT mice, n = 10 for middle-aged WT mice, n = 5 for old WT mice, n = 12 for middle-aged i-mn-*Sod1*^-/-^ mice and n = 10 for controls, and n = 7 for old i-mn-*Sod1*^-/-^ mice and n = 5 for controls.

### 4.2. Contractile Properties

The intact 1st lumbrical muscles were dissected from mice hind paws and transferred to a 0.25 mL chamber containing Tyrode’s solution (NaCl, 121 mM; KCl, 5.0 mM; CaCl_2_, 1.8 mM; MgCl_2_, 0.5 mM; NaH_2_PO_4_, 0.4 mM; NaHCO_3_, 24 mM; glucose, 5.5 mM; EDTA, 0.10 mM) maintained at 25 °C and perfused at a rate of 18 exchanges/min. The solution was bubbled with 95% O_2_, 5% CO_2_ to maintain oxygenation and pH 7.3. Lumbrical muscles were mounted horizontally in the chamber with one end attached to a stationary post and the other to a force transducer (modified Model 400A, Aurora Scientific, Inc., Aurora, ON, Canada). Parallel platinum plate electrodes on either side of the muscle were used for activation by electrical stimulation. Muscles were stimulated with single pulses of 0.2 ms duration to elicit twitch contractions. Stimulation voltage and muscle length were adjusted to elicit maximum twitch force. During twitch contractions, the time from the onset of force development to the peak of the force response was measured and reported as time to peak twitch tension (TPT), and the time for the force to decline by 50% from the peak is reported as the half relaxation time (HRT). Trains of stimulus pulses were administered at 125 pulses/s to generate maximum tetanic contractions. To calculate specific force, the maximum tetanic force was normalized by the cross-sectional area measured directly from immunofluorescent images used for the analysis of fiber type (see below).

### 4.3. NADH Fluorescence

The coenzyme nicotinamide adenine dinucleotide (NAD) is present in muscle fibers in both its reduced (NADH) and oxidized (NAD^+^) forms and the ratio of the two forms is critical to cellular respiration. NAD^+^ receives protons during glycolysis and the tricarboxylic acid (TCA) cycle, while NADH is oxidized during the electron transport chain to reform NAD^+^. NADH fluoresces when excited with ultraviolet (UV-A) light while NAD^+^ does not allowing NAD redox state to be monitored using fluorescence spectroscopy [63]. Intracellular NADH fluorescence is dominated by mitochondrial NADH [64], and total NAD (NAD^+^ and NADH) in the muscle does not change appreciably during the course of a given experiment. Thus, the NADH level relative to its full range reflects the mitochondrial redox status at any given point during the experiment.

To monitor muscle mitochondrial function in the context of normal physiological conditions, NADH fluorescence was recorded continuously before, during, and following a 5s tetanic contraction as previously described [8,19]. Briefly, the experimental chamber with polished quartz bottom was placed on the stage of an inverted microscope (Axiovert 100, Zeiss, Oberkochen, Germany). Fluorescence was elicited by epi-illumination from a 75 W xenon lamp and detected using a photomultiplier tube (model R1527, Hamamatsu Photonics, Japan). The wavelengths for excitation were centered at 361 nm, selected using a diffraction grating monochromator (DeltaRAM, Horiba Scientific, Kyoto, Japan). The emitted fluorescence passed through a 460 nm band-pass filter (50 nm bandwidth) before reaching the photomultiplier tube. Fluorescence responses were collected at the center of the muscle.

At the end of each experiment, maximum and minimum NADH levels for each muscle were determined by recording the fluorescence responses to the addition of sodium cyanide and carbonyl cyanide-4-(trifluoromethoxy)phenylhydrazone (FCCP), respectively. Specifically, the maximum NADH level was determined by replacing the chamber perfusion solution with Tyrode’s solution that included 5 mM sodium cyanide, an electron transport inhibitor. The introduction of cyanide resulted in a rapid increase in fluorescence that reached a plateau within 8–10 min. Basal fluorescence was quickly reestablished upon the reintroduction of normal Tyrode’s solution. Minimum NADH level was achieved by the introduction of a separate Tyrode’s solution to which FCCP (1 mM) had been added. Exposure to the mitochondria uncoupling agent for 60 min resulted in a decrease in fluorescence to a minimum plateau. All NADH fluorescence results are presented relative to the range between the minimum and maximum levels to account for differences in total NAD content.

### 4.4. Intracellular Calcium Transients

The dynamic calcium responses to twitch stimuli were performed as previously described [8,44]. Briefly, lumbrical muscles were incubated with the cell permeant acetoxymethyl (AM) ester form of the low-affinity calcium indicator mag-fura-2 (10 μM) (Catalog #M1292, Thermo Fisher Scientific, Waltham, MA, USA) for 30 min at room temperature. The Mag-fura-2 signal was excited by alternating between 344 nm and 375 nm and the emission signal was captured at 510 nm. Pre-loading backgrounds were subtracted from all signals, and the mag-fura-2 fluorescence ratio (344 nm/375 nm) was taken as an undistorted representation of the rapid intracellular calcium transient associated with a twitch contraction.

### 4.5. Immunofluorescent Staining

Lumbrical muscle cross-sections (10 μm) were used for staining different fiber types and longitudinal sections (25–35 μm) were used for staining neuromuscular junctions (NMJ) as described previously [8,65]. For cross-sections, lumbrical muscles were embedded in OCT compound and frozen in liquid nitrogen directly for 1 min and stored at −80 °C. Before sectioning, OCT embedded tissues were put at −20 °C, and sections were taken from the mid-portion of the muscle mounted on slides. For longitudinal sections, muscles were fixed in 4% paraformaldehyde for 4 min at room temperature. After washing with 0.01M PBS, tissues were cryoprotected with an increasing gradient of 20%, 30% sucrose. Then, muscle tissues were embedded in OTC (Triangle Biomedical Sciences, Durham, NC, USA) at −20 °C and cryo-sectioned into longitudinal sections and mounted on slides.

Sections were incubated in 0.01M PBS with 1% Triton X-100 containing 5% donkey serum for 30 min at room temperature to block non-specific binding sites. Muscles were incubated with primary antibody (Table 1) overnight at 4 °C. After washing well in 0.01 M PBS three times, muscles were incubated with secondary antibody and conjugated antibody (Table 1) for 1 h at room temperature. Tissues were then washed with 0.01 M PBS and a coverslip was placed over the sections with mounting medium. A Nikon confocal microscope was used for visualizing immunofluorescent signals. Quantitative analysis of NMJ structure and fiber types was performed using software associated with the Nikon confocal microscope (Tokyo, Japan) and ImageJ (Version 1.8.0). For analysis of NMJ morphology, at least 4 muscles were analyzed in each group and approximately 100 endplates were scored in each muscle. Depending on the extent of overlap of β-III tubulin with α-Btx, endplates were scored as fully innervated (100% overlap), partially innervated (10–80% overlap) or fully denervated [8,65].

### 4.6. Statistical Analysis

R 4.0.0 and Graphpad Prism 8.0.0 were used for statistical analyses. An unpaired student’s *t*-test for two groups and one-way or two-way ANOVA for more than two groups were used to generate *p* values. Pearson correlation analysis were used to measure the linear correlation of two variables. Quantitative data are presented as described in each figure, either as box plots including individual data points for each muscle analyzed along with minimum, first quartile (Q1), median, third quartile (Q3) and maximum numbers of the data set, or as bar plots with mean ± SEM. Outliers are identified as below Q1-1.5*(Q3-Q1) or above Q3+1.5*(Q3-Q1). Significance level is set at *p* < 0.05.

## 5. Conclusions

The current study showed that, during aging, impairments in skeletal muscle mitochondrial function precede the development of calcium handling defects and muscle weakness. In addition, alterations in redox homeostasis specifically in neurons are sufficient to accelerate muscle aging phenotypes, in the absence of frank denervation. Denervation-induced skeletal muscle atrophy has been proposed to occur at least in part through negative effects on mitochondrial function [13,66]; however, recent reports that muscle-specific deficiency of *Sod2* caused abnormalities at the NMJ indicate that there are likely bi-directional links between muscle mitochondria and NMJ maintenance [40]. The present observation that mitochondrial dysfunction was observed prior to evidence of a loss in innervation supports the possibility that mitochondrial dysfunction is a driver of NMJ degeneration and denervation. Our observation that neuron specific deletion of CuZnSOD was sufficient to induce mitochondrial dysfunction and calcium handling defects in the absence of denervation supports the conclusion that postsynaptic degenerative phenotypes observed in muscles of *Sod1*^-/-^ mice are not entirely secondary to NMJ degeneration. However, shifts in fiber type in i-mn-*Sod1*^-/-^ mice indicate remodeling of innervation and these transient denervation-reinnervation events may be sufficient to signal mitochondria. Finally, the acceleration of the fiber type shifts and mitochondrial dysfunction in i-mn-*Sod1*^-/-^ mice support the possibility that neuronal redox homeostasis is key for maintaining muscle function and its disruption is a major initiator of sarcopenia.

## Figures and Tables

**Table 1:** Antibodies Used for Immunofluorescent Staining.

Staining Target	Primary Antibody	Secondary Antibody
type 1 myosin heavy chain	BA-D5 (mouse IgG2b) (DSHB, Iowa City, IA, USA) (1/100)	Anti-mouse IgG2b 647nm, A21242 (Invitrogen, Carlsbad, CA, USA) (1/300)
type 2a myosin heavy chain	SC-71 (mouse IgG1) (DSHB, Iowa City, IA, USA) (1/100)	Anti-mouse IgG1 568nm, A21124 (Invitrogen, Carlsbad, CA, USA) (1/300)
type 2b myosin heavy chain	BF–F3 (mouse IgM) (DSHB, Iowa, USA) (1/100)	Anti-mouse IgM 488nm, A21042 (Invitrogen, Carlsbad, CA, USA) (1/300)
extracellular matrix		WGA CF405S conjugate, #29027 (Biotium, Hayward, CA, USA) (1/10) 1 h
axons and presynaptic terminals	βIII-tubulin (Rabbit, BioLegend, San Diego, CA, USA, 802001, 1/1000)	Anti-rabbit Alexa 488 (1:2000 Thermo Fisher Scientific, Waltham, MA, USA A11034)
acetylcholine receptors		Alexa Fluor 594-conjugated α-bungarotoxin (α-Btx; 1:2000; Thermo Fisher Scientific, Waltham, MA, USA B-13423)

## Data Availability

Most of the data are provided in this work. Other data that support the findings of this study are available from the corresponding author upon reasonable request.
